# Integrative Oncology Approaches to Supporting Immune Checkpoint Inhibitor Treatment of Solid Tumours

**DOI:** 10.1007/s11912-023-01492-4

**Published:** 2024-01-09

**Authors:** Nina Fuller-Shavel, Jonathan Krell

**Affiliations:** 1 Synthesis Clinic, Winchester, UK; 2 British Society for Integrative Oncology (BSIO), Midhurst, UK; 3 Oncio CIC, Stockbridge, UK; 4 Imperial College London, London, UK

**Keywords:** Immunotherapy, Immune checkpoint, Integrative oncology, Medical oncology, Diet, Microbiome

## Abstract

**Purpose of Review:**

The goal of this review was to examine the role and practical applications of integrative oncology strategies in supporting immune checkpoint inhibitor (ICI) treatment of adult solid tumours.

**Recent Findings:**

Beyond tumour-intrinsic factors, several patient-associated factors affect ICI response, including germline genetics, systemic inflammation, the gut microbiota, and diet. Current promising supportive interventions include a Mediterranean-style diet with over 20 g of fibre, regular exercise, use of live biotherapeutics, minimisation of PPI and antibiotic use, and ensuring vitamin D repletion, with many other integrative oncology approaches under study. Caution around medical cannabis use in patients on ICIs is advised due to previously documented adverse impact on overall survival, while VAE (*Viscum album* extract) therapy studies have not highlighted any safety concerns so far.

**Summary:**

With expanding ICI use, it is important to investigate and apply low-cost integrative oncology strategies to support better treatment outcomes and minimise adverse events. Further research may lead to pre-treatment assessment of both tumour and patient-associated biomarkers and personalised multimodal prehabilitation care plans, as well as on-treatment support with targeted nutrition, physical activity, and supplementation regimes, including both systemic inflammation and gut microbiome modulating strategies. Given the emerging understanding of chronic stress impact on ICI treatment outcomes, mind-body approaches require further investigation.

## Introduction—Immunotherapy and Immune Checkpoint Inhibitors (ICIs)

The immune system plays a protective role against tumorigenesis, and tumour progression is often associated with an exhausted or dysfunctional antitumour immune response [1]. Cancer cells can acquire the ability to evade detection by the immune system, a process that is mediated by upregulation of inhibitory molecules, such as immune checkpoints or immunosuppressive cytokines that restrict the antitumour activity of leukocytes. Therefore, immune inhibitory molecules have been identified as key therapeutic targets [2]. Immune-checkpoint inhibitors (ICIs) targeting such molecules (e.g. PD-1, PD-L1, or CTLA4) have been established as effective cancer treatments, particularly for endometrial cancer, melanoma, renal cell carcinoma (RCC) and non-small-cell lung cancer (NSCLC) [3]. ICIs aim to reverse tumour-induced immunosuppression by removing the suppression of cellular antitumour immunity induced by these checkpoints and their ligands, allowing reactivation of the antitumour immune response and subsequent clearance of tumour cells by the immune system. This can be associated with treatment-induced immune-related adverse events (irAEs), such as colitis, pneumonitis, and hepatitis, which are often mild but can be severe. These autoimmune or autoinflammatory manifestations can limit both the use and effectiveness of this therapeutic approach [1].

ICIs have shown efficacy as single agents or in combination. Furthermore, recent studies have demonstrated synergy of ICIs used in combination with other therapeutic modalities, such as the antiangiogenic agents bevacizumab or lenvatinib, and in combination with chemotherapies, such as carboplatin and paclitaxel in endometrial cancer [4, 5]. Other forms of immunotherapeutic approaches utilised in the cancer clinic include T-cell therapies, dendritic cells therapies, CAR-T cell therapies, and cancer vaccines.

Despite significant progress achieved with ICI use in several indications, including metastatic melanoma, ICI therapy response is highly variable between different tumour types and within specific patient populations for multiple reasons explored extensively in other literature [6, 7]. Predictive markers of response to ICIs include tumour-intrinsic parameters such as tumour mutational burden, microsatellite instability, baseline tumour cell PD-L1 expression, and the presence of tumour-infiltrating lymphocytes [1]. However, several tumour-extrinsic factors also affect response to ICIs including germline genetics, systemic inflammation, the gut microbiota, and diet. For example, recent studies have shown that the gut microbiome is a critical factor in determining response to ICIs, and immune activation appears to be functionally dependent on the patients’ gut microbial community and diversity [8–10]. In the search for strategies to enhance ICI response without excessive toxicity, integrative oncology offers promising avenues to modify systemic inflammation and the gut microbiome as some of the key mechanistic targets.

## Integrative Oncology—Whole Person Care in Supporting Treatment Outcomes

The BSIO (British Society for Integrative Oncology) defines integrative oncology (IO) way as a ‘patient-centred, evidence-informed field of cancer care that utilises psychological, nutritional, lifestyle and complementary interventions alongside conventional cancer treatments to support better quality of life, improve resilience, minimise the side effects of treatment and improve outcomes’ [11]. Integrative oncology transcends generic practices to provide a holistic and patient-centred perspective through dynamic evaluation and support for the physical, emotional, mental, and spiritual needs of individuals affected by cancer. IO complements core medical treatment plans throughout the cancer care continuum, from prehabilitation and in-treatment support to optimisation of survivorship care and living well with advanced cancer. IO strategies, from nutrition and lifestyle interventions to natural product use, have been studied in supporting a wide range of cancer treatments, including ICIs and targeted therapies [12••, 13, 14].

At its core, integrative oncology is deeply rooted in evidence-based medicine, leveraging the best available research evidence, clinical expertise, and patient values to formulate a rational, individualised, and comprehensive care strategy. Integrative oncology practice is supported by the Society for Integrative Oncology (SIO) Clinical Practice Guidelines, which provide comprehensive guidance for incorporating complementary and integrative therapies into conventional oncology clinical practice. The most recent guidelines include 2017 SIO ‘Clinical Practice Guidelines on the Evidence-based Use of Integrative Therapies During and After Breast Cancer Treatment’ [15] and two joint SIO-ASCO clinical practice guidelines on integrative medicine for pain management in oncology [16] and integrative oncology care of anxiety and depression in adults with cancer [17], with further guidelines in the pipeline. Next steps in IO research and potential guideline development may involve examining the intersection of precision and integrative oncology and the potential clinical impact of biomarker-guided yet whole person-oriented IO care plans.

## Systemic Biomarkers—Opportunity for Immunological Prehabilitation

Prehabilitation is now well-established as an important part of surgical management, providing multimodal pre-operative measures to improve functional capacity and support post-operative recovery [18, 19], with the recent ASCO guidelines specifically focusing on prehabilitation recommendations prior to lung cancer surgery [20]. However, the concept is equally applicable to any systemic anticancer therapy where there may be an opportunity for patient optimisation influencing treatment tolerability or outcomes.

Available research points to an association between poorer ICI treatment outcomes in multiple cancer types and elevated baseline levels of chronic inflammation, measurable by multiple indices, e.g. neutrophil-to-lymphocyte ratio (NLR), platelet-to-lymphocyte ratio (PLR), Lung Immune Prognostic Index (LIPI), and Systemic Inflammatory Index (SII) [21–25]. For example, LIPI, which consists of derived NLR and LDH, is the most studied score in advanced NSCLC with validated prognostic value in over five thousand NSCLC patients [21]. Another recent example from a sub-analysis of the INVIDIa-2 study showed that NLR (< 3.4) and SII (< 831) were independent prognostic indicators for OS in NSCLC, RCC, and melanoma treated with ICIs [23]. Higher baseline CRP levels have also been associated with poorer PFS and OS in multiple cancer types treated with ICIs, including NSCLC and melanoma [26–28]. Additionally, elevated cytokine levels, particularly IL-6 and IL-8, have been shown to play a prognostic role associated with shorter OS in ICI-treated patients but they are not easily measured in routine clinical practice [27, 29].

Instead of seeing inflammatory markers and associated indices purely as potential prognostic indicators or predictors of response, detecting baseline elevations may be an opportunity to optimise the patient’s chronic inflammation status. This may involve a multimodal approach, including anti-inflammatory nutrition interventions, regular exercise, reduction in risk behaviours (alcohol and smoking, with the latter being associated with an increased risk of ICI-related pneumonitis [30]), and where appropriate, exploring judicious use of appropriately timed supplements with anti-inflammatory or immunomodulatory action that do not have clinically significant interactions with patients’ medications [31–39]. While we have evidence on individual impact of different above named strategies on chronic inflammation levels [34, 38–42] with little associated risk, these approaches need formal evaluation in the immunotherapy prehabilitation setting, both alone and in combination.

Beyond baseline evaluation, following treatment initiation, inflammatory markers are promising predictive biomarkers of ICI response. For example, a CRP flare defined as a sharp CRP increase in the first weeks after starting treatment, followed by CRP decrease to below baseline, has been associated with ICI treatment response in NSCLC, head and neck cancer, metastatic urothelial carcinoma, and RCC [43–46]. This example illustrates appropriate immune response activation with increased likelihood of on-target anti-tumour impact without an excessive chronic inflammation tail. Combined with the data above, future approaches may look at modelling based on baseline and on-treatment inflammatory marker and index kinetics, initially within a predictive model and eventually within a model that encompasses targeted interventions at appropriate points in treatment, shifting prediction into responsive action.

In addition to inflammatory markers, we are seeing a building evidence base on the relationship between the gastrointestinal microbiome and ICI treatment response [47–49]. Future prehabilitation approaches can take advantage of the data available on nutrition highlighted below, including advising patients around consuming a Mediterranean-style diet with at least 20 g of fibre daily from a wide variety of plant sources [12••, 50••], and formal evaluation of such approaches in the pre-treatment setting would be a valuable addition to the evidence base.

As has been previously shown in the surgical prehabilitation and general integrative oncology setting, multimodal interventions delivered by an aligned multidisciplinary team are likely to have more impact on patient outcomes and quality of life [51–53]. Research that prioritises multidisciplinary programme evaluation in pre- and on-ICI treatment settings has the potential to have significant clinical impact.

## Physical Activity and ICIs

Physical activity and exercise have been shown to improve quality of life, reduce cancer-related fatigue, and reduce recurrence risk for certain cancers [20, 54–57]. However, we have a paucity of data on physical activity and ICI treatment impact with a few clinical studies, mostly focused on quality of life rather than combining these with oncological outcomes. In one retrospective study of 59 patients with unresectable hepatocellular carcinoma (HCC) on lenvatinib and anti-PD-1 antibodies, patients in the active group had significantly longer OS (HR = 0.220, 95% CI 0.060–0.799) and PFS (HR = 0.158, 95% CI 0.044–0.562) and higher ORR (OR = 4.571, 95% CI 1.482–14.102) than patients in the sedentary group [14]. Active patients were those who engaged with unsupervised training sessions at least five times per week of moderate aerobic activity for > 30 min or at least three days per week of vigorous aerobic activity for > 30 min/day or at least 3–5 days a week of mixed-intensity activity for more than 30 min/day before or within one month after the initiation of combination therapy. Two small prospective exercise programme pilots of 28–30 participants have also been completed, focusing on demonstrating feasibility with quality-of-life evaluation [58, 59]. Further clarity on the precise nature of effective exercise interventions and their impact on both oncological outcomes and quality of life may be provided with expected publication of further trials, including NCT04676009, NCT04263467, and NCT04866810. Until further data specific to immunotherapy is obtained, clinicians may consider recommending regular aerobic and resistance exercise during active treatment with curative intent as per ASCO guidelines [20] and reviewing available data on exercise for patients with metastatic disease, including bone metastases, for personalised recommendations [60–62].

## Role of Nutrition in Supporting ICI Treatment

Nutrition forms a core part of integrative oncology support with a broad impact on both cancer risk and clinical outcomes [63]. ESPEN (European Society for Clinical Nutrition and Metabolism) guidelines recommend the following key for action against cancer-related malnutrition, beyond obesity and cachexia management [64]:

- Screening of all patients with cancer for nutritional risk early in the course of their care, regardless of weight and BMI (body mass index) history
- Expansion of nutrition-related assessment practices to include measures of body composition, anorexia, inflammatory biomarkers, resting energy expenditure, and physical function
- Use of multimodal nutritional interventions with individualised plans, including focus on increasing nutritional intake, lessening inflammation and hypermetabolic stress, and increasing physical activity

Two important studies published in the last 5 years showed an association between dietary patterns and components and ICI outcomes. The first observational study of 128 patients with late-stage melanoma on ICI treatment was published in 2021, which showed that higher dietary fibre intake was associated with significantly improved progression-free survival (PFS) with the most PFS benefit for the group with sufficient fibre intake (20 g and above) and no probiotic use [50••]. Every 5g increase in daily dietary fibre intake was associated with a 30% lower risk of progression or death [50••]. This was followed by a multi-centre cohort study of 91 patients with advanced melanoma treated with ICIs where logistic generalised additive models revealed positive linear associations between a Mediterranean dietary pattern and the probability of ORR and PFS at 12 months [12••].

The limitation of these studies is that they are observational and have been confined to examining the effects on ICI treatment of advanced melanoma. Dietary fibre and Mediterranean diet (or other plant-rich anti-inflammatory diet) impact should be evaluated in other cancers and ideally within an interventional study design. Having acknowledged such limitations, provided there is no medical condition that would preclude a Mediterranean-style diet with at least 20 g of fibre daily, these are not difficult interventions to counsel patients around and may carry significant potential benefit that could extend beyond ICI support [65, 66]. Given the research above, our current pragmatic approach to supporting the gastrointestinal microbiome before and during ICI treatment makes the following recommendations within a personalised plan:

- Eating at least 20 g and ideally 30 g + fibre daily [50••, 66, 67] within the context of a Mediterranean-style diet [12••] or other culturally appropriate plant-rich anti-inflammatory diet
- Minimisation/avoidance of ultra-processed foods, processed meats and sugar-sweetened drinks that have been shown to adversely impact the gastrointestinal microbiome and systemic inflammation [68–73], with the latter being associated with poorer ICI treatment outcomes [21, 24]
  - Practically this often involves switching from a Western-style diet to a more prudent Mediterranean-style dietary pattern.
- Avoidance of antibiotics and proton pump inhibitors (PPIs) where possible due to their impact on the gastrointestinal microbiome [67, 74–76].

Future studies in this area may explore associations between ICI response and outcomes with DII (dietary inflammatory index)/E-DII (energy-adjusted DII) [77] and/or a variety of prudent dietary patterns appropriate to different cultural backgrounds, as well as looking at the intake of fermented foods and other nutritional components impacting the composition of gut microbiota and systemic inflammation, e.g. polyphenols [78–80].

## The Gut Microbiome and Supplementation—Live Biotherapeutics, Probiotics, Prebiotics, and Postbiotics

Immune activation appears to be functionally dependent on a patient's gut microbiome, and this is a critical factor in determining response to ICIs [8–10]. Furthermore, features of the gut microbiota have emerged as potential distinct biomarkers, and regulators of response to ICIs [8–10]. Clinical studies have utilised various strategies to modify the gut microbiome as a therapeutic approach. These have predominantly focused on faecal microbiota transplantation (FMT) or oral live biotherapeutics (LBTs). FMT constitutes a direct method of transferring a community of beneficial gut bacteria in faecal form from a donor to a patient. Baruch and colleagues performed FMT from melanoma patients who had a complete response to ICIs, to patients who had primary resistance to ICI therapy, and induced a 30% objective response rate in these ICI-refractory patients [81••]. However, FMT is very time and resource intense, and administration involves invasive procedures, such as a colonoscopy or nasogastric tube insertion [82]. Oral LBTs can be administered orally in capsule form, offering a much more desirable and patient-orientated approach to manipulation of the gut microbiome. Administration of several single-strain LBTs have been shown to enhance ICI response across a broad range of cancers. A pan-tumour neoadjuvant window study of a LBT, MRx0518, demonstrated long-term safety and clear evidence of immune modulation with significant anticancer efficacy [82]. In another example, *Clostridium butyricum* MIYAIRI 588 strain (CBM588) has demonstrated significant improvement in ORR and median PFS as a combination therapy in patients receiving nivolumab-ipilimumab and may buffer detrimental impact of PPIs in NSCLC patients receiving ICI therapy [83, 84]. LBTs have significant immune-modulatory effects through metabolites, such as short-chain fatty acids, enhancing response to ICIs clinically [85, 86]. Furthermore, LBTs have been shown to reduce irAEs, most notably of the gastrointestinal tract (e.g. colitis), when administered concurrently with ICIs [82, 85, 86].

The gut microbiota can be modulated by supplements such as prebiotics—non-digestible food ingredients that benefit the host by selectively stimulating the growth and activity of specific bacterial species in the colon. For example, inulin derived from chicory roots can promote the growth of colonic bacteria such as *Bifidobacterium*, which has been shown to promote immunosurveillance and response to ICIs [87].

Probiotics are live microorganisms, such as *Lactobacillus* and *Bifidobacterium* strains, that are intended to have health benefits when consumed or applied to the body. There are conflicting data on the effect of probiotic consumption and response to ICI, and their impact depends on the exact supplement strain being administered. Spencer et al. (2021) showed in clinical and pre-clinical studies that probiotic use was associated with reduced response to ICIs [50••]. However, Bender et al. (2023) demonstrated in mouse models that the probiotic *Lactobacillus reuteri* translocates to, colonises, and persists within melanoma tumours, and releases dietary tryptophan catabolite I3A that promotes interferon-γ-producing CD8 T cells within the tumour and bolsters response to ICI [88].

Postbiotics are metabolites or by-products secreted by live bacteria or released after bacterial lysis that have a physiological impact on the host. Short-chain fatty acids (SCFAs), such as butyrate and propionate, have been shown to drive CD4 and CD8 cell activation and their downstream effectors [89–91], enhance IFN expression and subsequently promote proinflammatory and anti-tumoural responses [92, 93], trigger apoptosis of cancer cells [94], and induce TLR-5-mediated NF-kB activation [95]. Lipopolysaccharides (LPS), O-antigen lipid-A, and 3-deoxy-D-manno-octulosonic acid generated by specific bacterial strains have been associated with inflammasome activation and with the secretion of the proinflammatory cytokines IL-1β, IL-18, TNFα, and IL-6 [89–95].

## Nutritional Supplements

Most of the research around ICIs and nutritional supplements has focused on vitamin D so far with its established immunomodulatory role [96]. A prospective study of 77 patients with advanced lung cancer showed that baseline 25-hydroxy-vitamin D levels were associated with ICI response and prognosis in terms of overall survival, with a relatively low baseline vitamin D level of 40 nmol/l and above carrying an AUC of 0.63 (*p* = 0.047) for partial ICI response [97]. This 25-hydroxy-vitamin D level would not be considered sufficient for osteoprotection where a minimum of 50 nmol/l is usually used a cutoff [98]. Interestingly, the prospective PROVIDENCE study of 164 patients with advanced cancer on ICIs used a more commonly used 75 nmol/l (30 ng/ml) serum 25-hydroxy-vitamin D cutoff. In this study, cohort 1, of whom 70% achieved adequate repletion with cholecalciferol supplementation at 3 months, showed longer overall survival (*p* = 0.013), time to treatment failure (*p* = 0.017), higher disease control rate (*p* = 0.016), significantly decreased risk of death (HR 0.55, 95% CI 0.34–0.90), and treatment discontinuation (HR 0.61, 95% CI 0.40–0.91) compared to cohort 2, all of whom were vitamin D deficient (< 75 nmol/l) [13]. Interestingly, cohort 1 patients also had a significantly decreased risk of all grade thyroid irAEs than the control cohort (OR 0.16, 95% CI 0.03–0.85) [13]. Future studies should focus on target level of a minimum of 75 nmol/l with potential investigation of higher serum target levels within the safe range (< 250 nmol/l) to assess the relationship in more detail.

Interestingly, there is a surprising lack of clinical studies around other common immunomodulatory or anti-inflammatory nutritional supplements, such as omega-3 and thymoquinone (derived from *Nigella sativa*, black cumin seed), which provides an excellent opportunity for future research. As another important example, mycotherapy (use of medicinal mushrooms) has promising preclinical evidence in supporting immunotherapy treatment [99], but this field needs investment into appropriate clinical research, ideally with multidisciplinary team and practising clinician involvement to guide dosing and administration schedules.

## Caution with Cannabis

Two main recent studies examined the association between medical cannabis use and ICI outcomes in adult patients with solid tumours. A prospective observational study of 102 patients with advanced cancers who initiated immunotherapy published in 2020 concluded that cannabis consumption, while reducing immune-related adverse events, also correlated with a significant decrease in time to tumour progression and overall survival [100••]. The second study published in 2023 examined 201 NSCLC patients on first-line monotherapy pembrolizumab, of which 102 commenced cannabis treatment, mainly for pain relief and loss of appetite. The group posited a ‘sigh of relief’ due to finding no significant difference in time to tumour progression and a non-significant OS difference (HR 95% CI 0.99 to 2.51, *p* = 0.08) [101]. However, it is important to note that, given the OS in the cannabis-naive group of 54.9 months versus 23.6 months in the cannabis-treated group and the available statistics above, the OS difference could have reached statistical significance with a larger sample size. Given the lack of consistent safety data outlined above, it is prudent for clinicians to avoid concomitant use of medical cannabis and ICIs until further research is conducted, ideally with more granularity around specific cannabis components that may influence ICI treatment, e.g. CBD vs CBG vs whole plant preparations as some examples.

## Chronic Stress and Potential for Mind–Body Therapies in ICI Support

Animal studies have shown that chronic stress with continuous activation of the hypothalamic–pituitary–adrenal (HPA) axis and the sympathetic nervous system (SNS) unfavourably alters the TME (tumour microenvironment) in a way that could promote tumorigenesis, invasion, progression, metastasis, and attenuation of systemic therapy effects, including anti-PD-L1 immunotherapy [102–104]. Two recent human studies have shown an adverse impact of pre-treatment emotional distress and chronic stress on ICI treatment outcomes in melanoma and NSCLC [105]. In a post hoc analysis of the phase 2 PRADO trial, pre-treatment emotional distress was associated with reduced major pathologic responses and 2-year recurrence-free and distant metastasis-free survival after neoadjuvant ICB treatment in patients with stage IIIB-D melanoma [105]. In the STRESS-LUNG-1 trial, chronic psychological stress was the independent prognostic factor for ORR (HR = 3.93; *p* < 0.001), PFS (HR = 1.59; *p* = 0.038), and OS (HR = 3.16; *p* = 0.005) in stage IIIB–IV NSCLC patients treated with first-line ICI therapy [106]. As the use of mind–body therapies, such as yoga, Tai Chi, acupuncture, and other modalities, for stress management has not been formally evaluated in the context of ICI treatment support, this is an important area for further clinical research.

## Other Integrative Oncology Therapies

VAE (*Viscum album* extract) or mistletoe parenteral administration has a rich history of use in integrative oncology and much ongoing research interest. As an immunomodulatory agent with some immunostimulatory properties, there were historical concerns around VAE use concomitant with ICIs. The first small clinical safety study on combining VAE with nivolumab, ipilimumab, or pembrolizumab in 16 patients with advanced or metastatic cancer showed no difference in adverse event rates, including irAEs, between ICI only and ICI + VAE groups [107]. A further real-world observational cohort study of 242 breast and gynaecological cancer patients showed that there was no difference in the safety profile of targeted therapy administration alone vs combination group, although only 13 patients in the total cohort received ICIs [108]. This is perhaps not surprising, given immunotherapy use was largely confined to triple negative breast cancer (TNBC) within the breast cancer group and limited to specific gynaecological cancer indications. While current data on VAE + ICE combinations is limited, it is consistent in not demonstrating a safety signal and is in line with our clinical experience. Therefore, provided VAE use is recommended and supervised by an experienced qualified integrative physician, it may be used with careful monitoring. Further studies in common ICI populations, e.g. melanoma and NSCLC, are needed with subcutaneous VAE to clarify the risk–benefit profile.

Herbal medicine from multiple traditions is a commonly used immunomodulatory approach in integrative oncology, with CHM (Chinese Herbal Medicine) currently being the most studied herbal approach in general oncology support. Chinese herbal medicine approaches have promising preclinical evidence in supporting immunotherapy treatment [109, 110], and the results of a planned multi-centre RCT evaluating the safety and efficacy of Bojungikki-tang (BJIKT) therapy in patients with advanced NSCLC treated with ICIs are eagerly awaited [111].

## Conclusions

Immune checkpoint inhibitor (ICI) therapy has made a significant clinical impact in several solid tumour types. However, given response variation, associated expense, and irAE impact, it is important to investigate and apply low-cost integrative oncology strategies that could support better treatment outcomes and minimise adverse events. As research in this area progresses, it may lead to pre-treatment assessment of both tumour and patient-associated biomarkers and personalised multimodal prehabilitation care plans, as well as on-treatment support with targeted nutrition, physical activity, and supplementation regimes, including both systemic inflammation and gut microbiome modulating strategies.

**Fig. 1:**
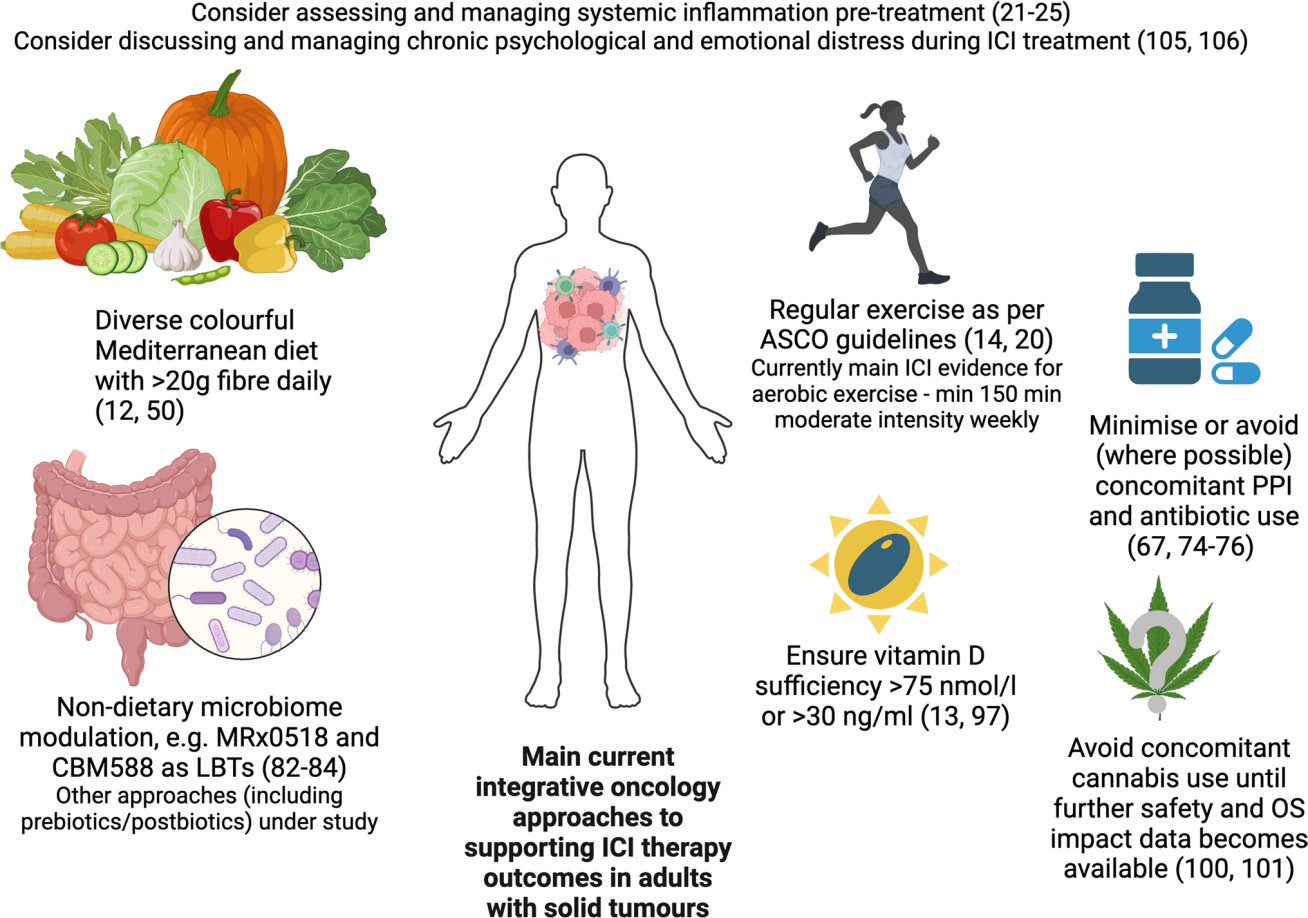
Main current integrative oncology approaches to supporting ICI therapy outcomes in adults with solid tumours (created with BioRender.com)

Integrative oncology (IO) offers promising avenues for modifying systemic inflammation and the gut microbiome as some of the key strategies to enhance ICI response without excessive toxicity. The main current IO approaches in ICI support outlined in Fig. 1 include a Mediterranean-style diet with over 20 g of fibre [12••, 50••], regular exercise [14], the use of live biotherapeutics [82–84], ensuring vitamin D repletion [13, 97], and minimising PPI and antibiotic use [67, 74–76], with many other integrative oncology approaches under study. This area is ripe for further research, ranging from dietary intervention and dietary component studies, e.g. polyphenols, prebiotics, and omega-3, to further exploration of diverse gut microbiome and immune modulation approaches, from mycotherapy to Chinese herbal medicine.

Caution around medical cannabis use in patients on ICIs is advised due to previously documented adverse impact on overall survival [100••, 101], while VAE (*Viscum album* extract) therapy studies have not highlighted any safety concerns so far [107, 108]. Given the emerging understanding of chronic stress impact on ICI treatment outcomes [105, 106], it is prudent to discuss stress management with patients, with mind–body modality use in ICI support being an important area for further investigation.

## Data Availability

No datasets were generated or analysed during the current study.
